# P02.81. Symptom management with massage in postoperative colorectal patients: a randomized controlled trial

**DOI:** 10.1186/1472-6882-12-S1-P137

**Published:** 2012-06-12

**Authors:** N Delzer, B Bauer

**Affiliations:** 1 Mayo Clinic, Rochester, USA

## Purpose

We analyzed the impact of post-operative massage in patients who had undergone abdominal colorectal surgery.

## Methods

A randomized-controlled trial was designed for colorectal abdominal surgery patients. Patients were randomized to either receive a 20 minute massage on POD 1 and 2, 20 minutes of ambulation, or a visit by the massage therapist (no massage). Baseline physiologic data (HR, SBP/DBP, RR) were collected 30 minutes prior to and after either intervention. A standardized assessment of patient perception of pain, tension, anxiety, satisfaction with overall care, and relaxation level utilizing a visual analog scale (10 point Likert scale) was obtained before and after the intervention. The IRB approved study was powered to detect a difference at the 80% level. Repeated measures models were used to compare the symptom scores at different time points. T-tests were used to compare same day pre-post intervention changes.

## Results

128 patients (62 massage [M], 66 control [C]) were randomized. There was no statistical difference between the groups in regards to age, gender, diagnosis, history of prior abdominal surgery, or mode of surgery (open vs. laparoscopic). Over the first two post-operative days, M patients experienced a significant reduction in anxiety (p=0.01) and improvement in relaxation (p=0.003) but not in pain (p=0.15), tension (p=0.11), or satisfaction (p=0.21) as compared to C patients. The only physiologic variable altered was a reduction in diastolic BP (p=0.04) in the M group. A comparison between baseline, prior to any intervention, and after intervention on POD1 demonstrated the M patients experienced a reduction in pain, tension, anxiety, and an improvement in relaxation relative to C patients. The same holds on POD2 pre-post intervention.

## Conclusion

In a randomized controlled trial of patients undergoing abdominal colorectal surgery, we demonstrated that post-operative massage resulted in a significant improvement of patient’s perception of post-operative pain, tension, and anxiety.

